# The Concise Language Paradigm (CLaP), a framework for studying the intersection of comprehension and production: electrophysiological properties

**DOI:** 10.1007/s00429-024-02801-8

**Published:** 2024-05-15

**Authors:** Natascha Marie Roos, Julia Chauvet, Vitória Piai

**Affiliations:** 1 Donders Center for Cognition, Radboud University, Postbus 9104, Nijmegen, 6500 HE Netherlands; 2 Max Planck Institute for Psycholinguistics, Nijmegen, Netherlands

**Keywords:** Spoken word production, Language comprehension, ERPs, Alpha, Beta, Prediction

## Abstract

Studies investigating language commonly isolate one modality or process, focusing on comprehension or production. Here, we present a framework for a paradigm that combines both: the Concise Language Paradigm (CLaP), tapping into comprehension and production within one trial. The trial structure is identical across conditions, presenting a sentence followed by a picture to be named. We tested 21 healthy speakers with EEG to examine three time periods during a trial (sentence, pre-picture interval, picture onset), yielding contrasts of sentence comprehension, contextually and visually guided word retrieval, object recognition, and naming. In the CLaP, sentences are presented auditorily (constrained, unconstrained, reversed), and pictures appear as normal (constrained, unconstrained, bare) or scrambled objects. Imaging results revealed different evoked responses after sentence onset for normal and time-reversed speech. Further, we replicated the context effect of alpha-beta power decreases before picture onset for constrained relative to unconstrained sentences, and could clarify that this effect arises from power decreases following constrained sentences. Brain responses locked to picture-onset differed as a function of sentence context and picture type (normal vs. scrambled), and naming times were fastest for pictures in constrained sentences, followed by scrambled picture naming, and equally fast for bare and unconstrained picture naming. Finally, we also discuss the potential of the CLaP to be adapted to different focuses, using different versions of the linguistic content and tasks, in combination with electrophysiology or other imaging methods. These first results of the CLaP indicate that this paradigm offers a promising framework to investigate the language system.

## Introduction

A complete understanding of the language system in the brain requires a characterization of that system in its fullest range. However, studies investigating language usually isolate one modality, focusing on either comprehension or production (e.g., for comprehension, fMRI: Binder et al. 2000; EEG: Boulenger et al. 2011; for production. fMRI: Rizkallah et al. 2018; EEG: Rabovsky et al. 2021 (EEG). It is also worth noting that the modality predominantly examined in neuroimaging studies is comprehension, rather than production. For example, in two meta-analyses on language production and comprehension in fMRI, the studies included on production amounted to at most one third of those included for comprehension (Indefrey, 2018; Walenski et al., 2019). In the present study, we propose a framework to study both aspects of language simultaneously, by tapping into comprehension and production within the same trial: The *Concise Language Paradigm* (CLaP). Furthermore, we combined the CLaP with electroencephalography (EEG), given it provides direct measures of net neuronal activity with excellent temporal resolution, which is helpful for tracking the time-course of brain activity during speech comprehension or production.

The attempt to investigate language comprehension and production together is not novel, as illustrated by studies investigating language processing during conversation (Bögels 2020). In this study, a turn-taking setting in face-to-face interviews was used in combination with EEG. Findings revealed that the start of response planning was accompanied by a positive ERP peak as soon as enough information had been revealed in the question. On average, this happened after only one third of the question had been asked, suggesting that planning of production largely overlaps with comprehension. As another example, others have used naturalistic language to compare spontaneous production and comprehension by means of syntactic processing (Giglio et al. 2024). Participants were asked to produce or listen to spontaneous speech, both combined with fMRI. Results revealed different time-courses for the sensitivity to syntactic structures in production and comprehension, suggesting an anticipatory or integratory approach, respectively. The relationship between language production and comprehension specifically has also been discussed in more detail (Meyer et al., 2016). Although these are good examples of how one can successfully combine the study of language production and comprehension, they do not exactly provide room for systematicity to manipulate different aspects within a paradigm. That is, the paradigms are not set up as a *framework* that can be adapted to different focuses and thereby potentially serve several purposes. This is, in fact, the aim we pursued in the present study.

**Fig. 1:**
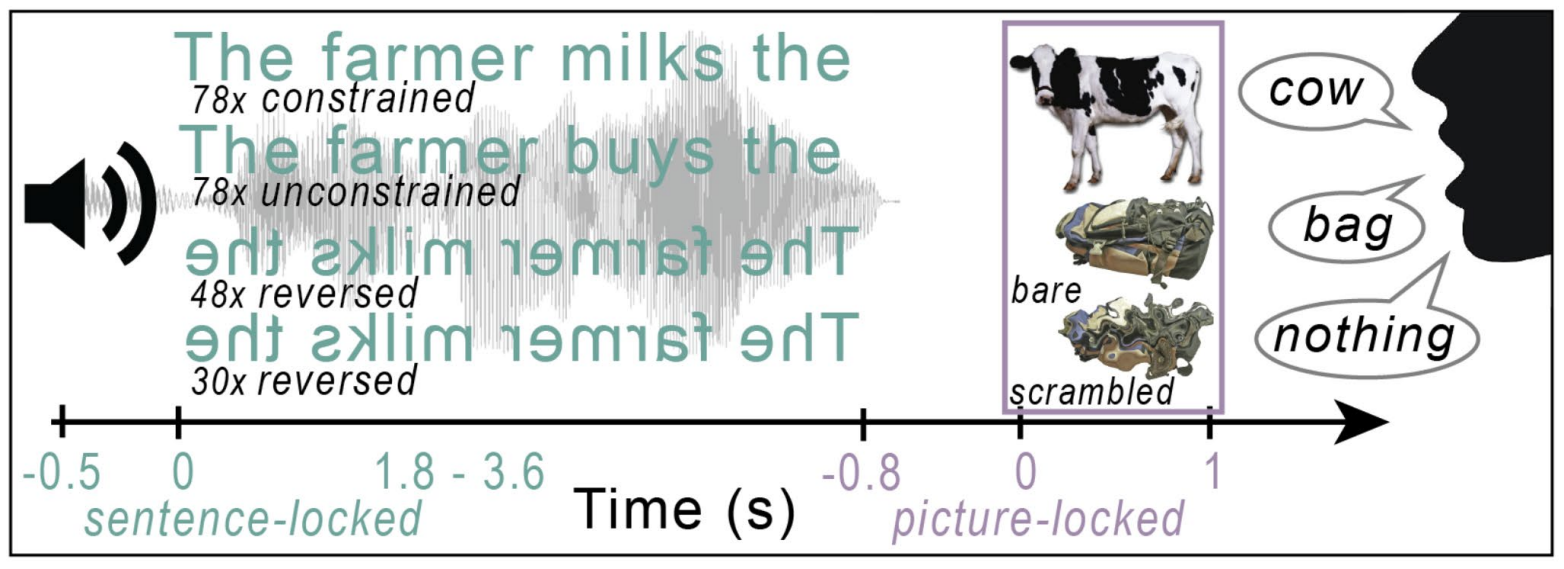
Schematic overview of example trials for each condition and total amount of trials per condition (78, 78, 48, 30, respectively, in the order presented). The different sentence conditions are constrained, unconstrained, and reversed, while picture types are normal or scrambled. This yields the following naming conditions: constrained and unconstrained naming (i.e., saying “cow”), bare naming (i.e., saying “bag” always following reversed sentences), and scrambled naming (i.e., saying “nothing” always following reversed sentences). Note the different time-lockings color-coded to either sentence or picture onset on the time-line. Sentence time varied per trial from 1.8–3.6 s. Reproduced with permission from the authors from 10.17605/OSF.IO/W5Q7S

In the CLaP, all trials have exactly the same structure across conditions. This means they require the same instructions to participants and reduce task-related confounds between conditions as much as possible, as participants do not have to switch between performing different tasks. Irrespective of condition, all trials present an auditory stimulus followed by a visual stimulus. Specifically, participants listen to sentences that are either contextually constrained towards the last word, unconstrained, or time-reversed. The last word of constrained and unconstrained sentences is shown as a picture, which participants have to name. Time-reversed sentences are either followed by a normal picture, which participants also name, or a scrambled picture, which participants name with a stereotypical utterance (e.g., “nothing”). Fig. 1 illustrates the paradigm and the trial structure per condition. As such, the CLaP yields several low-level and high-level contrasts of language processes that are part of comprehension as well as production, such as sentence comprehension, contextually and visually guided word retrieval (Roos et al. 2023), object recognition, and naming.

The present study provides a characterization of the electrophysiological properties of the CLaP, focusing on three different time periods within a trial. For that, we derived a (somewhat) simplistic measure of language comprehension/speech perception by comparing sentence-locked brain responses to meaningful speech sentences with those of unintelligible time-reversed speech (i.e., a low-level auditory control condition). In addition, we attempted to clarify the nature of the context effect, a neurophysiological effect associated with pre-activation of information following constrained sentences, but preceding the picture. Finally, we examined object recognition and naming by comparing (early) picture-locked brain responses for normal objects (as a function of the preceding context) with those of meaningless scrambled objects (i.e., a low-level visual control condition), on the one hand, and picture naming times across these conditions, on the other hand. Below, we briefly review previous studies that are relevant for the contrasts reported here. Some of these contrasts are known for yielding phenomena at the level of event-related potentials (ERPs), and others at the level of oscillations (which include both phase-locked and non-phase-locked activity), quantified by time-frequency-resolved modulations (TFRs). We follow a chronological order of the trial structure throughout (i.e., sentence comprehension, pre-picture context effect, object recognition and naming following picture presentation).

### Sentence comprehension

Time-reversed speech is an unintelligible counterpart of real speech with the same physical complexity and global acoustic characteristics. While real speech tends to have fast onsets and long decays, reversed speech results in the opposite, yielding sound sequences that do not occur in real speech. Reversed speech thus conveys less phonetic and lexical-semantic information and is commonly used as a non-semantic control condition for auditory speech processing (Binder et al. 2000; Narain et al. 2003; Stoppelman et al. 2013; for electrophysiology Brown et al. 2012; Forseth et al. 2018). When used in combination with fMRI, some studies report stronger BOLD responses in bilateral superior temporal regions to reversed speech (Binder et al. 2000; but see Brown et al. 2012), while other studies report that the BOLD responses of reversed speech mostly overlap with those of real speech in frontal and temporal language regions, arguing that it provides a less optimal baseline to isolate speech processing regions (Narain et al. 2003; Stoppelman et al. 2013). In terms of ERPs, it has been found that signal amplitude is increased by time-reversal of speech relative to intelligible speech in the first 300ms of stimulus presentation (Boulenger et al. 2011). In the present study, this could lead to a difference in auditory evoked ERPs (and their counterpart in the time-frequency domain) for reversed versus real speech.

### Context effect

The context effect in the pre-picture interval has been investigated in several previous studies. In the electrophysiological signal, the contrast between constrained and unconstrained picture naming yields power decreases in the alpha-beta frequency range (8–25 Hz) prior to picture onset (e.g., Piai et al. 2014, 2015a, b, 2017, 2018). This effect is generated in left hemisphere (posterior) perisylvian areas and is not only replicable across studies, but also across sessions within-participants (Klaus et al. 2020; Roos and Piai 2020; for fMRI see Roos et al. 2023). These power decreases have been interpreted as processes of word retrieval taking place before picture onset in constrained, but not in unconstrained picture naming. In fact, we found that the amount of power decreases correlates with picture naming times, such that faster picture naming is associated with stronger power decreases in constrained picture naming (Roos and Piai 2020). On the contrary, there was no correlation between these power decreases and cloze probabilities (i.e., percentage of people who complete the sentence with the correct target word) for constrained contexts (Hustá et al. 2021).

These results suggest that the context effect is more directly related to picture naming processes, rather than being aspects of sentence comprehension. However, the interpretation of these pre-picture alpha-beta power decreases has remained unsubstantiated so far by the lack of a control condition. That is, it remains unclear whether the relative power decreases in constrained versus unconstrained picture naming might, in fact, emerge from power *increases* in unconstrained picture naming, rather than power *decreases* in constrained picture naming. Thus, while we expected to replicate the context effect, the addition of time-reversed speech trials as a low-level auditory control condition to the CLaP would further clarify the underlying power dynamics of this effect.

### Object recognition and naming

Finally, we evaluated object recognition and naming in the context of the current CLaP conditions. That is, whether object recognition and naming are modulated by the meaningfulness of the object (i.e., normal, scrambled) and the type of sentence preceding the picture (i.e., constrained, unconstrained, reversed). For that, we examined both the brain responses and the naming times of correctly named targets.

Bare and scrambled picture conditions, both preceded by reversed sentences, differ in the extent to which they engage language production stages. While scrambled pictures are all named with the same stereotypical utterance (“nothing”), normal picture naming requires all stages, which predicts faster naming for scrambled pictures relative to normal pictures. Regarding brain responses to visual stimuli, previous studies have found differences in ERP amplitudes for scrambled versus normal objects. Visual evoked potentials (VEPs) for scrambled pictures showed higher amplitudes compared to normal objects over posterior sites (Gruber and Müller 2005). Another study investigating brain network modularity for normal and scrambled pictures found higher interaction between brain modules for scrambled compared to normal pictures in visual processing brain regions (Rizkallah et al. 2018). They interpreted this finding as increasing communication in the brain while trying to match the characteristics of the scrambled pictures to already existing representations in visual memory. As the scrambled pictures are unknown and cannot be recognized as existing objects, all such attempts fail. With regard to the present study, these findings predict higher amplitudes in response to scrambled pictures compared to meaningful pictures (in the absence of a priming lead-in context).

In visually guided naming, the concept is presented to participants as a picture, whereas in contextually guided naming it emerges from the semantics of the constrained sentences. Accordingly, previous studies have shown faster naming times for constrained pictures relative to unconstrained pictures (Griffin and Bock 1998; Piai et al. 2014). This suggests that, for constrained naming, a concept and its associated label (i.e., “lemma”), and potentially even a corresponding phonological form are already pre-activated (Piai et al. 2014, 2020) before the picture is visually presented. This could lead to a different brain response at picture presentation for constrained pictures compared to all other conditions, where no concept (and lexical and phonological information) is activated yet. However, the best-controlled contrast to test this hypothesis is the comparison between picture naming following constrained versus unconstrained sentences, as both are preceded by meaningful speech stimuli.

Other studies on picture naming have linked the P2 component (approx. 200ms post picture onset) to processes of lexical selection (Fargier and Laganaro 2017, 2020; Indefrey 2011), including associating it with the ease or difficulty of lexical selection. These studies report positive correlations between ERP amplitudes and picture naming times, such that high P2 amplitudes are associated with less accessible words and slower naming, and lower P2 amplitudes with more easily accessible, high-frequency words and faster naming (Rabovsky et al. 2021; Strijkers et al. 2010). This would suggest lower amplitudes for constrained pictures around 200ms after picture onset, as lexical information is retrieved prior to picture onset in constrained picture naming.

Finally, if naming that is not primed by a sentence is also not hindered by it, naming following unconstrained sentences should not be any different from commonly used bare picture naming. Both are visually guided naming conditions, but preceded by different auditory sentence stimuli (unconstrained: meaningful, bare: reversed). In a previous single case study of a person with aphasia due to extensive left hemisphere damage, the rate of anomia was the same for unconstrained and bare picture naming, whereas constrained sentences increased the rate of successful naming attempts (Chupina et al. 2022). This led us to predict no difference between these two different types of visually guided picture naming in the current study.

### Summary

In sum, for comprehension, we anticipated a difference in brain responses to time-reversed speech compared to real speech after sentence onset. We further expected to replicate the context effect between constrained and unconstrained picture naming, where power in the alpha-beta frequency range is decreased before picture onset. By virtue of the time-reversed speech sentences, we hoped to clarify whether this effect is driven by power decreases following constrained or power increases following unconstrained sentences. This clarification would add to our understanding of the context effect and how oscillatory brain activity can be mapped onto retrieval processes. Finally, we predicted higher ERP amplitudes and faster naming for scrambled pictures compared to meaningful ones. As word retrieval in constrained picture naming starts prior to picture presentation, we expected the most divergent ERP amplitudes after picture onset and fastest responses for this condition. Finally, regarding visually guided naming, we expected no differences in ERPs or naming times for unconstrained and bare picture naming. Collectively, these hypotheses aim to provide an understanding of the brain responses underpinning the current version of the CLaP, and highlight the paradigm’s approach to integrate measures of comprehension and production within a single trial.

## Methods

This study was approved by the Ethics Committee of Social Sciences at Radboud University, following the Code of Ethics of the World Medical Association (Declaration of Helsinki). Informed consent was obtained from all individual participants included in the study. Neither the study nor any procedures or analyses were pre-registered prior to conducting the research. Data collection took place at the Donders Centre for Cognition (Radboud University) in Nijmegen in the Netherlands. The data and code are available via the Donders Repository (10.34973/19gn-7v46*).*

### Participants

We recruited 26 participants in total (20 females) between the ages of 18 and 28 (*M* = 22, *Mdn* = 24) to participate in the study for monetary compensation. One participant was a substitute for an unusable dataset due to missing EEG markers, another four datasets were excluded due to measurement mistakes (2) and noisy EEG data (2). Thus, the data presented here derives from a total of 21 subjects (15 females), for which we report naming accuracy and EEG results. For two subjects, there were no audio recordings available, so naming time results are based on 19 subjects. All participants were right handed, native speakers of Dutch, with normal or corrected-to-normal vision and normal hearing, and without any neurological or language deficits.

### Materials

The experimental stimuli consisted of 156 pictures to be named by the participants. While 126 of those were photographs of objects depicting the target noun, 30 pictures were scrambled pictures which are meaningless and unknown to the viewer, used as a low-level visual control condition. Photographs were taken from the BOSS database (Brodeur et al. 2010) and from the internet. Scrambled pictures were created based on photographs from the BOSS database and distorted to make them unrecognizable without majorly changing the basic visual properties, called diffeomorphic transformation (Stojanoski and Cusack 2014). All object or scrambled pictures were depicted on black background with a height of 270 pixels. Seventy-eight of the photographs were preceded by an auditorily presented sentence that was either constrained or unconstrained towards the target noun. Sentence recordings were taken from a previous study (Chupina et al. 2022), recorded by a native speaker of Dutch at a slow pace. Sentences were 4 to 8 words long including the target word (M = 6 words) and auditory sentence duration varied from 1.79 to 3.59s (M = 2.61s, Mdn = 2.62s). The cloze probabilities for the target words were 0–39% in unconstrained sentences and 60–100% in constrained sentences (*t*(77) = 51.236, *p* < .001) (Chupina et al. 2022; Hustá et al. 2021). The other 48 photographs for bare picture naming and the 30 scrambled pictures were preceded by a reversed speech sentence. The reversed speech stimuli were created by time-reversing the 78 shortest constrained and unconstrained sentences using in-house MATLAB code.

### Design

The experiment consisted of 234 trials in total. Sentences were divided into three different conditions: constrained (*n* = 78), unconstrained (*n* = 78), and reversed (*n* = 78). Constrained and unconstrained sentences preceded the same 78 pictures, such that each of these pictures appeared once after a constrained and once after an unconstrained sentence. The reversed sentences preceded either a normal photograph of an object for bare picture naming (*n* = 48), or a scrambled picture as control (*n* = 30). Thus, there were four production conditions: constrained naming, unconstrained naming, bare naming, and scrambled naming. Every trial was set up in the same manner: audio followed by visual stimuli to be named, irrespective of condition. Trials were pseudorandomized using Mix (van Casteren and Davis 2006) with at least 20 trials between both appearances of the same target picture and no more than four consecutive trials of the same condition, yielding a unique list per subject. Figure. 1 illustrates example trials of each condition.

### Procedure

Before giving informed consent we instructed participants about the EEG measurement and the experimental task. The purpose was to listen to the auditory stimuli and accurately name the following picture with the respective target noun. We instructed participants to name scrambled pictures with the word “niks” (Dutch for *nothing*). During the EEG preparation, participants went through all photograph stimuli and the corresponding target nouns in a slide show, including some examples of scrambled pictures, to decrease naming variability and increase accuracy. Before the start of the EEG recording, we instructed participants to sit as still as possible, keep their back and shoulders relaxed and to keep blinking to the blinking intervals following picture offset. Participants sat on a chair in front of the computer screen on which the experiment was presented. We presented the stimuli by means of Presentation software (*Neurobehavioral Systems Inc*., Berkeley, CA, www.neurobs.com*)*, which enabled us to start audio recordings with picture onset to capture participants’ responses for later analyses. Each trial started with a fixation cross in the middle of the screen and the presentation of the auditory stimulus after 1500ms. Then, 800ms after auditory stimulus offset the respective picture appeared on screen for 1000ms, followed by a 2000ms blinking interval showing three asterisks (***), leading to the following trial. Before the start of the EEG recording we started with eight practice trials (three normal sentences and five reversed sentences) to ensure that participants had understood the task well. If not, the practice trials were repeated. The experiment was divided into nine blocks of 26 trials each and lasted around 40 min. After each block, participants could determine if and for how long they wanted to rest. A whole testing session including preparation and EEG recording took approximately 120 min.

### EEG acquisition

The EEG was recorded continuously using BrainVisionRecorder from 64 active electrodes. Electrode positions were based on the international 10–20 convention using an Acticap system and a BrainAmps DC amplifier (500 Hz sampling, 0.016–100 Hz band-pass). The online reference electrode was placed on the left mastoid and the ground electrode in the position of “AFz”. Impedance for both was kept below 5 kOhm, and below 10 kOhm for all other electrodes. The vertical and horizontal electro-oculogram (EOG) were recorded with electrodes above and next to the eyes, and an additional electrode (“T7”) removed from the cap to be placed below the left eye. Two other electrodes (“T8”, “TP10”) were removed from the cap to be placed on the orbitalis muscle on the right side of the mouth, above and below the lips. To synchronize the presentation of the stimuli with the EEG data, we sent condition specific markers at the onset of every sentence and picture which were recorded with the EEG data.

### EEG preprocessing and analyses

EEG preprocessing and analyses were performed in MATLAB using FieldTrip (Oostenveld et al. 2011). For data cleaning, we cut the trials from 800ms before sentence onset to 1400ms after picture onset. Trials were demeaned and low-pass filtered at 50 Hz. After rejecting incorrect trials, we visually checked the data for flat and noisy channels, or trials to be removed due to excessive artifacts (except eye movements and blinks). Then we performed an Independent Component Analysis (ICA) to remove components from the data that were related to blinking and eye movements (Jung et al. 2000). After ICA, we interpolated bad channels using a weighted average of all neighboring channels and then re-referenced the data to the common average of all channels. Finally, we visually inspected the data and marked the remaining noisy segments in each trial.

All EEG analyses were conducted on the scalp level, locked either to sentence or picture onset. Sentence or picture segments containing artifacts marked during visual inspection of the preprocessing stages were discarded from the analyses. Segments for sentence-locked analysis were cut from − 800ms to 1800ms relative to sentence onset. Note that this time point is based on the length of the shortest included sentences. Hence, for the longest meaningful sentences this segment only included approximately the first half of the sentence. For picture-locked data, the segment was − 1000ms to 300ms relative to picture onset.

All contrasts were calculated based on the highest common number of available trials across conditions per comparison. Excess trials were removed by sentence length to meet this common number (shortest sentences included), as reversed sentences were created based on the 78 shortest meaningful sentences. For the sentence-locked analyses, all conditions (constrained, unconstrained, reversed) included a maximum of 78 trials. The pre-picture context contrasts (TFRs) were based on a maximum of 48 trials (amount of bare picture trials); and picture-locked ERPs on a maximum of 30 trials (amount of scrambled picture trials).

All ERPs were low-pass filtered at 40 Hz and averaged across trials per participant and condition. Sentence-locked ERPs (78 trials) were baseline-corrected using the interval from − 800ms to sentence onset, while picture-locked ERPs (30 trials) were not baseline-corrected, as the signal amplitude was modulated by the preceding sentences resulting in systematic differences in “baseline” periods. All TFRs were computed for frequencies from 3 to 40 Hz with a sliding time window of 3 cycles, advancing in steps of 50ms and 1 Hz. Each time window was multiplied with a Hanning taper with implemented time–frequency transformation based on multiplication in the frequency domain. TFRs and ERPs were then averaged across trials per participant and condition.

#### Statistical analyses

For sentence comprehension, we compared meaningful and reversed speech sentences, serving as a (somewhat) simplistic measure of language comprehension/speech perception. Here we looked at sentence-locked ERPs for all three conditions to investigate auditory evoked potentials after sentence onset. We also looked at TFRs for the three possible contrasts between conditions, both from − 800ms to 1800ms relative to sentence onset.

For the context effect, we looked at TFRs by contrasting the 800ms pre-picture interval for constrained and unconstrained picture naming, as well as constrained versus reversed and unconstrained versus reversed sentences. Here we only used sentences preceding bare picture naming (48 trials), to ensure that this time interval is not affected by anything related to scrambled picture naming. We also looked at the context effect based on all (max 78) constrained and unconstrained trials per participant. As a final sanity check, we compared the pre-picture intervals of bare and scrambled picture naming (both preceded by reversed sentences).

For the object recognition and naming analyses, we compared picture-locked ERPs across conditions. As the scrambled condition contained only 30 trials per session, all picture-locked ERP comparisons are based on a maximum of 30 trials. We were specifically interested in the comparisons of scrambled versus bare picture naming (difference in picture stimuli), constrained versus unconstrained picture naming (difference in sentence constraint), and unconstrained versus bare picture naming (difference in preceding auditory-speech stimuli).

All ERP as well as TFR contrasts were evaluated by means of non-parametric cluster-based permutation tests (Maris and Oostenveld 2007) on the group-level. A dependent samples t-test was performed at every channel-time(-frequency) sample and those exceeding a threshold (*p* < .05, two- tailed) were identified for subsequent clustering (adjacent time and frequency samples, minimum number of neighboring channels = 2). A cluster-level statistic was defined as the sum of t values within each cluster. Then, based on 1,000 random permutations of the conditions being tested, the same clustering procedure was performed. The Monte Carlo p-value of the empirically observed clusters was computed as the amount of 1,000 random permutations yielding a more extreme cluster-level statistic than the observed one, again at an alpha level of 0.05 (two-tailed). All available time (and frequency) points and channels entered the comparisons.

### Naming time analysis

We coded participants’ picture naming responses online during the EEG recording for accuracy. If participants hesitated, uttered more than one word or nothing at all, responses were coded as incorrect. Synonyms for the target nouns were coded as correct, provided that they made sense in the context of the preceding sentence, if applicable. Voice recordings started with picture onset and lasted for 3500ms. Based on these recordings we later manually marked the naming times using the speech editor PRAAT (Boersma and Weenink 2017), blinded for condition. Statistical analysis of the naming times was done in R (R. Core Team 2017) for correct trials only. To get all relevant comparisons between contrasts, we ran two linear mixed-effects regression models. Both models had fixed effects of condition and by-participant and by-item random intercepts and slopes for condition. The reference used for the first model was bare picture naming to which the other conditions were compared. For the second model, we used constrained picture naming as the reference, to have a direct comparison of naming times for constrained picture naming with unconstrained and scrambled picture naming.

## Results

On average, participants (*n* = 21) made between 0 and 5 errors in naming (M = 2, Mdn = 2, SD = 1.7). The overall error rate was very low, 0.9% (43 errors in total). Participants made no errors in naming scrambled pictures, 10 errors in bare picture naming (0.2%), 13 in constrained picture naming (0.3%), and 20 in unconstrained picture naming (0.4%). All results below comprise trials with correct naming responses. For the discussions of scalp topographies in any of the results reported below, we would like to refer the reader to the limitation Sect. 4.5, where we discuss the interpretability of such EEG topographies in more detail.

### Sentence comprehension (sentence-locked ERPs and TFRs)

**Fig. 2:**
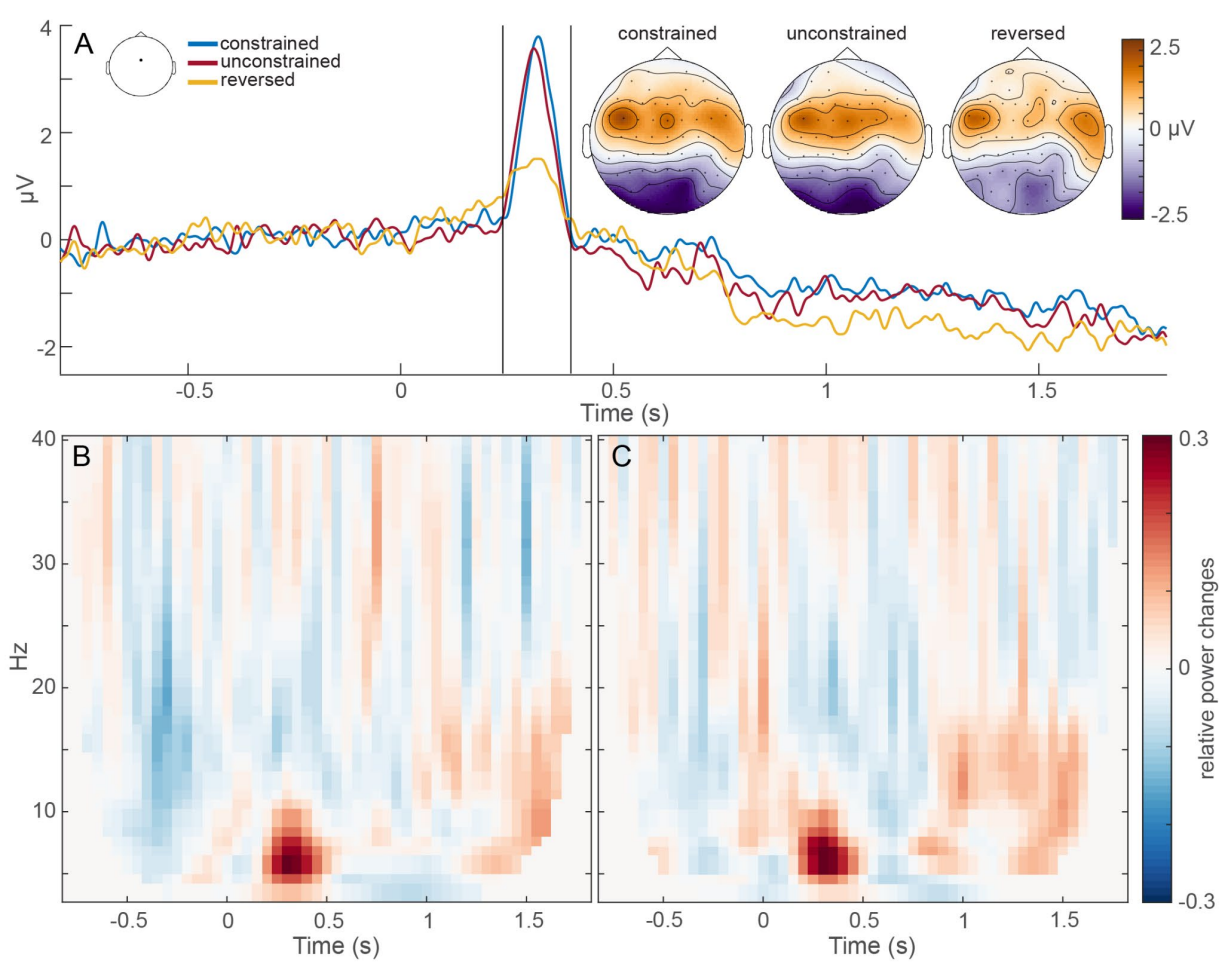
Event-related potentials (**A**) and time-frequency representations (**B**, **C**) during sentence onset (-0.8 to 1.8s) for electrode FCz as shown in the empty topographic schematics on top. Topographic plots show topographies in µV for the ERPs from 240-400ms after sentence onset as marked by the two vertical lines in the ERP plot per condition. Time-frequency plots in panels B and C display relative power changes (difference between conditions divided by their mean) for the difference between constrained and reversed (**B**) and unconstrained and reversed sentences (**C**). Time 0s represents sentence onset in all plots. Reproduced with permission from the authors from 10.17605/OSF.IO/W5Q7S

As a (somewhat) simplistics measure of comprehension, we compared ERPs across sentence conditions (constrained, unconstrained, reversed). These results are shown in Fig. 2A. The responses to the auditory speech stimuli were clearly visible for constrained and unconstrained sentences (both meaningful speech) from 240ms to 400ms after sentence onset with a peak amplitude between 3.5-4µV for electrode FCz (for electrode location see Fig. 2A). These potentials both significantly differed from reversed speech (ps < 0.006), which also showed a slight amplitude increase in the same time-frame, but with a much lower peak amplitude of 1.5µV. We found no significant differences between constrained and unconstrained sentences.

In Fig. 2A, we also show the topographies for the auditory response peak from 240-400ms per condition. In constrained and unconstrained sentences, we see a strong posterior negativity and a central to bi-lateral positivity. In reversed sentences, the amplitude is smaller in general, and the positivity seems to be more focal and symmetrical, rather than central. This could potentially indicate the difference between left-lateralized processing of speech in meaningful sentences compared to low-level auditory, non-linguistic input in reversed sentences, but this interpretation would require confirmation with source-level analysis.

Following this initial evoked response, reversed sentences continued to diverge from meaningful speech with sustained amplitude differences throughout the course of the sentence, significantly differing from unconstrained sentences between 1.1 and 1.4s (*p* = .0099).

To investigate oscillatory dynamics during the sentence time-window (-800ms to 1800ms), we looked at all possible contrasts between the three sentence types. The most salient aspect of the TFRs of both constrained and unconstrained relative to reversed sentences was the power increase up to 10 Hz corresponding to the phase-locked responses we see in the ERPs 240-400ms after sentence onset, as shown in Fig. 2B and C. Constrained relative to unconstrained sentences did not yield any significant differences in this interval, neither did the contrast between unconstrained and reversed sentences. The only significant difference in TFR contrasts during the sentence was between constrained and reversed sentences, which yielded one cluster of power increases (*p* = .032).

### Context effect (pre-picture TFRs)

**Fig. 3:**
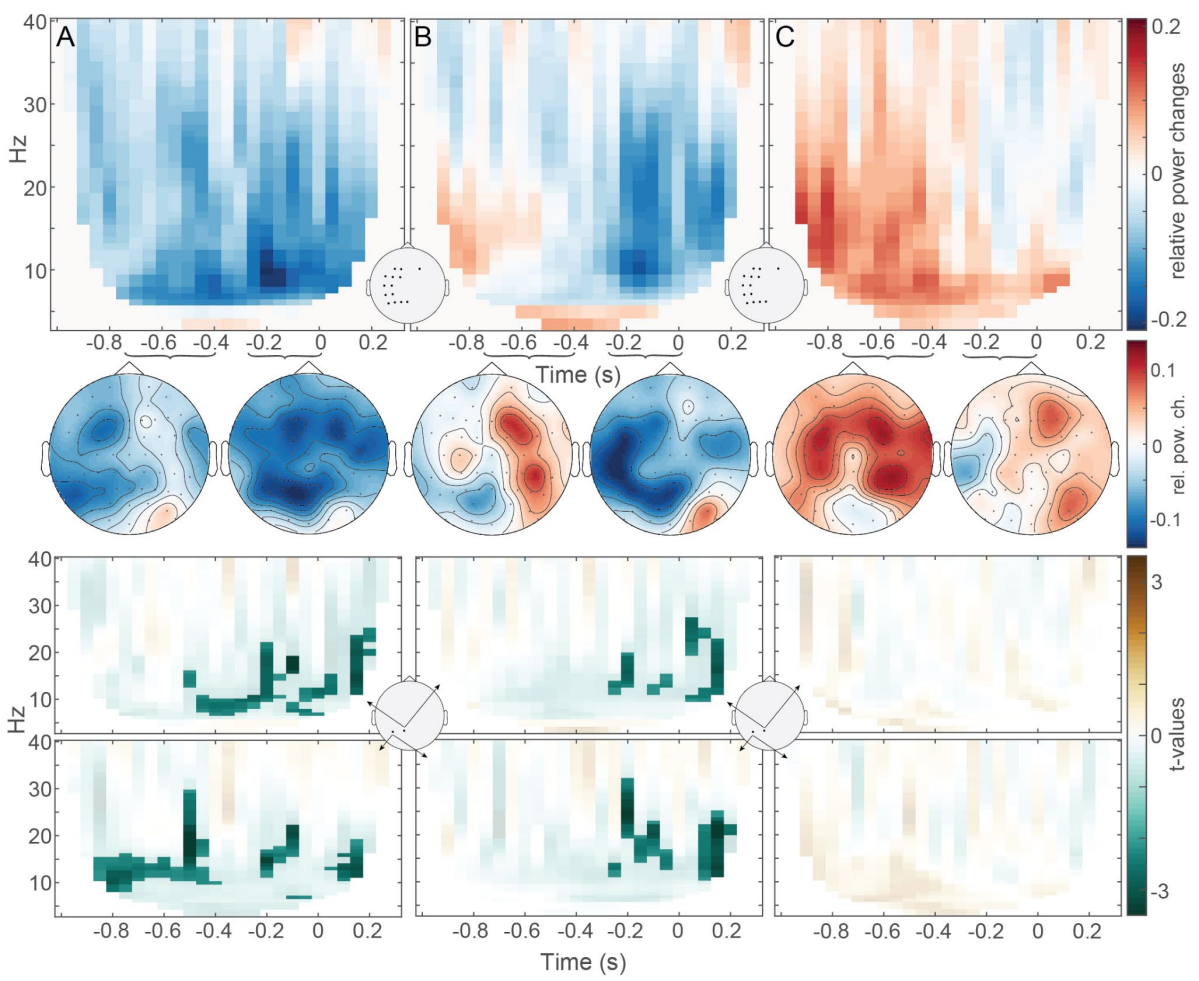
Time-frequency representations during the pre-picture interval per contrast between conditions. All TFR effects were calculated by taking the difference between two conditions of interest divided by their mean. Time 0s represents picture onset, -0.8s is sentence offset. (**A**) constrained relative to unconstrained sentences, (**B**) constrained relative to reversed sentences, (**C**) unconstrained relative to reversed sentences. Selected channels are shown in the empty topographic plots in between. The large topographic plots show topographies from 8–25 Hz for − 750ms to -400ms (left topographic plot) and − 250ms to 0ms (right topographic plot) per contrast. The lower TFR plots show t-values masked at the cluster-level threshold for channels P1 (top) and P5 (bottom). The contrast of unconstrained relative to reversed sentences (**C**) does not yield any significant clusters during the pre-picture interval for the selected channels. Note that the upper two colorbars show relative power changes and the lower one shows t-values. Reproduced with permission from the authors from 10.17605/OSF.IO/W5Q7S

Contrasting constrained and unconstrained pre-picture intervals yielded the expected context effect as found in previous studies. The most prominent cluster of power decreases was found in the alpha-beta frequency range of approximately 8–25 Hz, prior to picture onset, spanning the entire time-window from − 800ms to 200ms relative to picture onset (*p* = .002). The power decreases initially have a left bias in the topographies, but become more widespread over time. These results, together with time-frequency plots of the t-values above cluster-level threshold for two different channels, are depicted in Fig. 3A. The same pattern was found when all available trials per participant (78 instead of 48) were used in the comparison.

So far, the nature of the context effect has remained underdetermined. In theory, the power differences could derive from power increases in unconstrained picture naming, rather than power decreases in constrained picture naming. To investigate this, we contrasted the pre-picture intervals following both meaningful sentence types with those following the low-level control condition of reversed sentences. Just as for constrained versus unconstrained sentence contexts, this analysis revealed significant clusters of power decreases for constrained versus reversed pre-picture intervals (ps ≤ 0.03). The respective topographies show initial power increases around sentence offset over right electrode sites, as well as left-hemisphere power decreases becoming more dominant and widespread before picture onset (see also the lower two plots with t-values above cluster-level threshold before as well as after picture onset, Fig. 3B).

The comparison of unconstrained and reversed pre-picture intervals resulted in a significant cluster of power increases around sentence offset (*p* = .04), which can be seen in Fig. 3C. Here, the topographies almost exclusively reveal central to bilateral power increases just after sentence offset that become weaker towards picture onset with a slight left lateralized decrease prior to picture onset. For this contrast (unconstrained versus reversed pre-picture intervals), we do not get any t-values above threshold at the same two electrodes that yielded above-threshold t-values for the previous two comparisons. For our sanity check of comparing the pre-picture intervals of bare versus scrambled picture naming, both preceded by reversed sentences, the results revealed no significant differences between these two conditions.

In sum, we find similar alpha-beta power decreases for both contrasts including constrained picture naming, opposed to power increases for unconstrained picture naming at sentence offset. This indicates that the context effect is characterized by power decreases linked to constrained picture naming, rather than (only) power increases during unconstrained sentences or before unconstrained picture naming.

### Object recognition and naming

#### Picture-locked ERPs

**Fig. 4:**
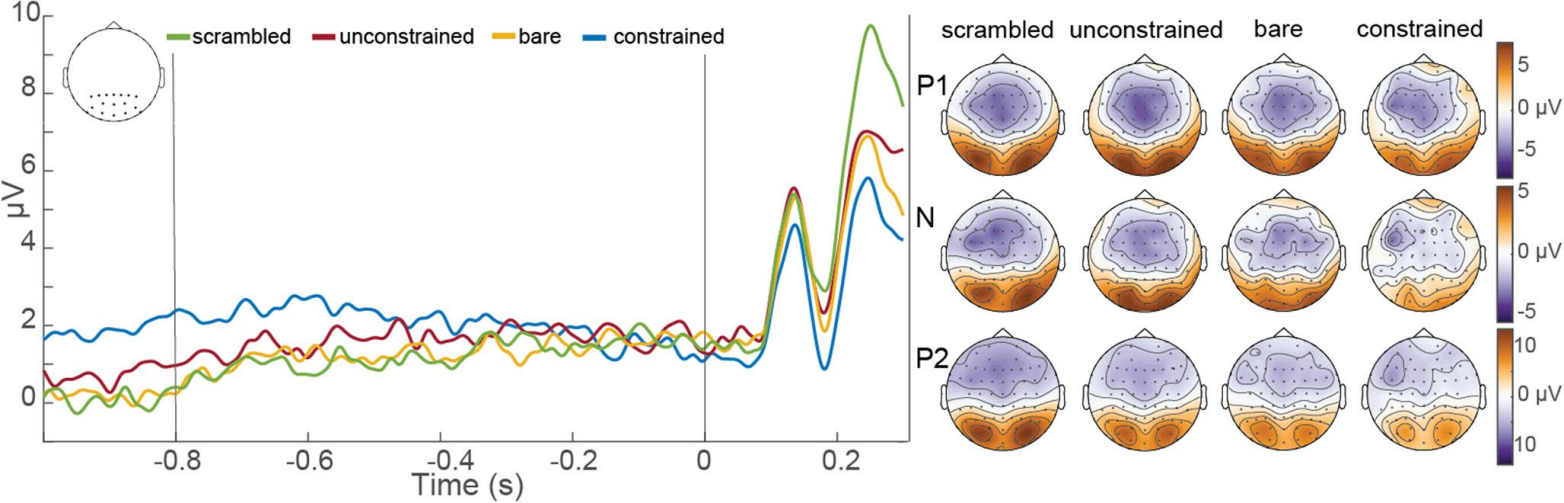
Event-related potentials during pre-picture interval and picture presentation per condition over selected occipital channels (shown in the empty topographic plot in the top left corner). The first vertical line at -0.8s represents sentence offset and the second vertical line at 0s represents picture onset. For each peak of the visual evoked potential (P1: 100-160ms, N: 160-200ms, P2: 200-300ms) we show the respective topography in columns per condition on the right. The color scales are the same per ERP component, but different between components. Reproduced with permission from the authors from 10.17605/OSF.IO/W5Q7S

In order to compare ERPs after picture onset across conditions, and specifically visual evoked potentials (VEPs), we investigated the potentials locked to picture onset. Based on the data as plotted in Fig. 4 for occipital channels, we divided the three different components of the VEP as follows: first positive component (P1) at 100-160ms, the following negative component (N) at 160-200ms, and second positive component (P2) at 200-300ms. The pattern of condition-specific VEPs remains the same, also when plotting different channel groups than occipital.

In the right half of Fig. 4 we show the corresponding topographies to the P1, N, and P2 (rows) per condition (columns). These clearly show strong occipital responses to the visual stimuli, in line with visual evoked potential topographies in other studies (cf. Gruber et al. 2004; Gruber and Müller 2005). The topographies of constrained sentences diverge most from the other three conditions, which is in line with the ERP results showing lower amplitudes across all three components.

Firstly, we evaluated general object recognition and naming by comparing bare with scrambled picture conditions. As both are preceded by reversed sentences, they only differ in the aspect of showing a normal picture (bare) versus a scrambled picture. During the pre-picture interval, the ERPs of bare and scrambled pictures do not show any difference. Only after picture onset their VEPs diverge. While the P1 still looks the same for both, bare picture naming has a significantly lower amplitude in the N component (*p* = .002), as well as the P2 component (*p* = .002).

To examine the assumption that word retrieval in constrained picture naming already starts prior to picture presentation, we compared the ERPs between constrained and unconstrained picture naming. As demonstrated in Fig. 4, the ERPs of constrained and unconstrained pictures already significantly differ during the end of the sentence and first half of the pre-picture interval, from − 1000 to -500ms (ps < 0.034), indicating that the ERPs are already modulated by the degree of sentence constraint. With respect to the VEPs of constrained and unconstrained picture conditions, they differ in all three components (100-300ms), with constrained sentences showing significantly lower amplitudes at all three potentials (ps < 0.004).

Finally, we also investigated visually guided naming, for which we compared bare and unconstrained picture naming (preceded by reversed and meaningful sentences, respectively). The ERPs of these two conditions behaved very similarly throughout the VEPs, as well as the rest of the segment, and only significantly differed from − 540 to -440ms during the pre-picture interval (*p* = .034). The amplitudes of their VEP components are highly similar throughout, and only start to diverge towards the end of the segment, after the P2.

#### Naming times

**Fig. 5:**
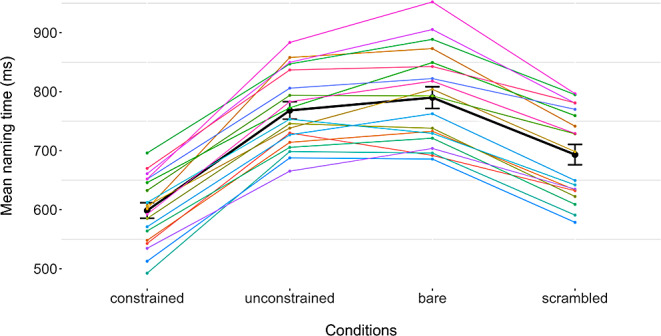
Mean naming times per condition. All values for one participant are connected by one line. Black dots connected by the black line represent the group mean per condition with error bars representing the standard error of the mean per condition. The slope of lines between two dots represents the effect size between these two conditions. Reproduced with permission from the authors from 10.17605/OSF.IO/W5Q7S

The mean naming times per condition on the group level were 599ms for constrained picture naming (Mdn = 602, SD = 57), 693ms for scrambled picture naming (Mdn = 698, SD = 75), 768ms for unconstrained picture naming (Mdn = 753, SD = 64), and 790ms for bare picture naming (Mdn = 793, SD = 8), based on 19 participants. These naming times are shown in Fig. 5A, with a different color for every participant. The slope of the lines connecting two dots represent the effect size between these two conditions (that is, the steeper the line, the bigger the effect). Figure. 5B displays the group mean per condition as well as the standardized error of the mean.

The first linear mixed effects regression to compare naming times (in ms) per condition with bare picture naming as the reference showed that bare picture naming was significantly slower than constrained picture naming (191ms avg. difference, Estimate = -192.07, SE = 11.69, t = -16.43, *p* < .001), as well as scrambled picture naming (97ms avg. difference, Estimate = -97.47, SE = 14.83, t = -6.57, *p* < .001). For bare and unconstrained picture naming there was no significant difference (22ms avg. difference, Estimate = -22.08, SE = 11.7, t = -1.89, *p* = .06). For the second model, we used constrained picture naming as the reference. Unsurprisingly, this yielded significant differences between constrained picture naming and all other conditions: unconstrained (169ms avg. difference, Estimate = 169.99, SE = 5.03, t = 33.79, *p* < .001), scrambled (94ms avg. difference, Estimate = 94.6, SE = 13.68, t = 6.91, *p* < .001), bare (Estimate = 192.07, SE = 11.69, t = 16.43, *p* < .001).

The naming time results are well aligned with the differences observed in the ERPs after picture onset, shown in Fig. 4. Bare and scrambled picture naming (both preceded by reversed sentences) differed significantly in naming times. Regarding constrained picture naming, these results confirm that processes of word retrieval must start before picture onset, as constrained picture naming was significantly faster than all other conditions. Especially in comparison to scrambled pictures, which were only named with a stereotypical utterance instead of having to retrieve a specific word presented by the picture, and should, therefore, have been very fast. For visually guided naming, the comparison of bare and unconstrained picture naming revealed no significant difference, although bare and unconstrained picture naming differ in the type of sentence preceding the picture (reversed versus unconstrained). These results show that it makes no difference for picture naming whether the preceding sentence contains real or reversed speech, unless the real-speech sentence is constrained towards the target word.

#### Summary

Taken together, the picture-locked results show that the ordering of conditions in terms of ERP amplitudes and naming times does not follow from the type of sentence preceding the picture (constrained, unconstrained, reversed). Rather, the key factors modulating behavior and signal amplitude are, firstly, that of a real object being presented, and secondly, the preceding sentence context. The semantic context in constrained sentences leads to conceptual pre-activation and word retrieval prior to picture onset (context effect), which is absent in all other conditions, regardless of whether their lead-in sentence was reversed or meaningful (unconstrained).

## Discussion

The present study was conducted to test the new Concise Language Paradigm, or CLaP, a framework designed to tap into contrasts of language comprehension as well as production within a single trial. The current version of the CLaP combines a (what we admit to be simplistic) measure of sentence comprehension, contextually and visually guided word retrieval (Roos et al. 2023), object recognition, and naming. Here, we focused on three different time periods within a trial, comprising a low-level contrast of sentence comprehension compared to reversed speech, the nature of the context effect prior to picture onset between constrained and unconstrained sentences, as well as object recognition and naming of normal and scrambled objects in different contexts.

With regard to sentence comprehension, we found that ERPs around sentence onset differed between reversed and meaningful speech, but not between constrained and unconstrained sentences. As expected, we replicated the well-established context effect of alpha-beta power decreases prior to picture onset in constrained versus unconstrained picture naming (e.g., Piai et al. 2014; Roos and Piai 2020) and could clarify its nature: the effect arises from power decreases for constrained picture naming, rather than power increases for unconstrained picture naming. Early brain responses after picture onset revealed differences in the N and P2 components for bare and scrambled pictures, both following reversed sentences. Also, contextually and visually guided picture naming differed in terms of their ERPs, with reduced ERP amplitudes for constrained pictures compared to the other conditions. The ERPs of visually guided naming, by contrast, were not modulated by whether the preceding sentences were unconstrained or time-reversed. Finally, picture naming was fastest for constrained naming, followed by scrambled picture naming, and equally fast for bare and unconstrained picture naming. Below, we discuss the three different time periods in more detail.

### Sentence comprehension

The current measure of sentence comprehension was based on a comparison of brain responses to the auditory sentence stimuli (constrained, unconstrained, reversed). The evoked potentials after sentence onset occurred in the same time frame across conditions, namely from 240 to 400ms (see Fig. 2). The peaks of constrained and unconstrained sentences hardly differed, because both are meaningful speech and there is no lexical-semantic difference between conditions yet at that point. The peak of reversed sentences, however, was relatively lower. This is in line with previous findings describing an early decay of the brain signal due to top-down attenuation once the auditory input is classified as non-speech (Stoppelman et al. 2013).

But not only the first evoked potentials differ between meaningful and unintelligible speech. After the initial evoked response, reversed sentences also diverged from meaningful speech with sustained lower amplitude than constrained and unconstrained sentences. Apart from the strong power increases in the sentence TFRs (corresponding to the ERP peaks after sentence onset), one could argue that the TFRs of real speech relative to reversed speech start to show power increases after sentence onset, increasing towards the end of time window (see Fig. 2B and C). This is in fact in line with sentence offset (which corresponds to the beginning of the pre-picture interval in Fig. 3), where both real speech conditions show power increases before and after sentence offset relative to our baseline condition (see Fig. 3B and C). This suggests that listening to real speech relative to reversed speech stimuli is characterized by power increases during sentence comprehension. However, our sentence stimuli as well as the analysis approach we adopted here were likely not optimized for studying the condition-specific differences during the sentences in more depth. Given our proposal of the CLaP as a framework to study language comprehension and production, one could easily opt for a different analysis strategy, a point to which we turn below.

### Context effect

We replicated the context effect of power decreases between constrained and unconstrained sentences in the alpha-beta frequency range before picture onset over left-hemisphere channels, associated with word retrieval (Piai et al. 2014; Piai, Roelofs, Rommers, & MariPiai et al. 2015a, b, 2017, 2018, 2020). This effect is already visible just after sentence offset, if not earlier, and intensifies towards picture onset.

Adding reversed speech as a low-level baseline condition to the paradigm enabled us to further characterize the dynamics of the power decreases during the pre picture interval following constrained and unconstrained sentences. Previous studies without such a control condition could not inform us about the direction of alpha-beta power changes for constrained and unconstrained sentences relative to the same baseline. Here, we demonstrated that power in the alpha-beta range decreases towards picture onset when preceded by constrained sentences. While power did seem to increase after unconstrained sentences, these power increases happened more around sentence offset rather than towards picture onset as in constrained sentences, and thus suggest to be a characteristic of real speech sentences relative to reversed speech, as discussed above in Sect. 4.1.

Thus, we can capture the alpha-beta power context effect even without contrasting with unconstrained sentences directly, as done in previous studies. In fact, contrasting constrained sentences with a simple low-level auditory baseline in the form of reversed speech sentences also revealed the context effect in the present study.

### Object recognition and naming

Our results for the comparison of ERPs locked to picture onset clearly showed that VEPs differed as a function of condition. While all conditions showed the same peak timings of the VEP (P1: 100-160ms, N: 160-200ms, P2: 200-300ms), their amplitudes differed. However, the way in which they diverged from each other did not follow from the sentence type preceding picture onset (constrained, unconstrained, reversed).

If we take visually guided picture naming (bare and unconstrained pictures) as the norm, one could argue that the only divergence appears in scrambled and constrained picture naming, due to aspects of visual appearance and sentential semantic constraint, respectively. Finally, we replicated the behavioral facilitation in naming times between constrained and unconstrained sentences from previous studies (Klaus et al. 2020; Roos et al. 2023; Roos and Piai 2020). We discuss the different conditions in more detail below.

#### Scrambled pictures

The VEP amplitude during the P2 component had a much higher amplitude for scrambled pictures compared to normal pictures. This is in line with previous studies investigating ERPs in response to scrambled pictures (Gruber and Müller 2005), where it has been interpreted as the integration of an unknown visual stimulus trying to be matched to an existing object in visual memory (Rizkallah et al. 2018).

Although these previous findings would explain the current VEP results for scrambled pictures, we think that picture naming times for scrambled objects also need to be considered. These were significantly shorter than those of bare picture naming and, in fact, the shortest after constrained picture naming. Participants did not have to comprehend and process a sentence preceding the picture and only had to identify the stimuli as scrambled, that is, recognize that the picture is not depicting a real object. Then they could directly name the scrambled picture with the Dutch high-frequency word for *nothing* (“niks”).

Potential integration processes of matching unknown visual stimuli to visual memory as suggested in the literature have not kept our participants from naming scrambled pictures faster than normal pictures without context. We thus argue that the high amplitude in VEPs of scrambled pictures might also be driven by seeing an unknown and therefore highly unexpected stimulus. Since only 30 out of 234 trials (< 13%) presented scrambled pictures, participants likely did not familiarize themselves with this type of stimuli. To compare, a standard oddball paradigm usually uses an oddball stimuli appearance of about 20%.

Previous research has linked the P2 component of VEPs to the ease of lexical selection (Rabovsky et al. 2021; Strijkers et al. 2010). This explanation alone, however, cannot directly account for our results. Scrambled pictures evoked the highest P2 amplitude, but revealed the second shortest picture naming times across conditions, being named with the high-frequency word “nothing”, which should be easily accessible. Therefore, the high P2 amplitudes in response to scrambled pictures in our study might simply be due more to visual than lexical aspects, whereas the modulations of the P2 component for real objects could indeed be explained by ease of lexical selection (see below). Thus, it should not be disregarded that the P2 component in picture naming studies is also the response to a visual stimulus.

Attention has previously been mentioned as a potential confound of the P2 effects in word production (Strijkers et al. 2010). Larger P2 amplitudes for rare or salient compared to normal stimuli would again be in line with our argumentation for the high P2 amplitudes in response to scrambled pictures in our study. In another study including similar control conditions to ours, scrambled pictures were also named significantly faster than normal pictures (Forseth et al. 2018). Altogether, these findings are an important reminder that it may sometimes be difficult to draw a one-to-one mapping between an ERP component and a cognitive process (e.g., the P2 component in word production uniquely reflecting lexical selection).

In sum, object recognition and stereotypical naming for scrambled pictures is faster than for real pictures. However, the visual stimuli are new to the viewer and thus likely elicit high VEP amplitudes, especially during the P2 component.

#### Constrained pictures (contextually guided naming)

Another interesting aspect of the VEP differences across conditions is the picture appearance after constrained sentences. Here, the amplitude is the lowest of all four conditions for all three components. This suggests that early brain responses to picture presentation are modulated by the pre-activation of a concept and associated information induced by sentence constraint. The extent to which not only language-related processes, but also (more low-level) visual processes are involved in this reduction of the amplitude, as found in repetition suppression (Gruber et al. 2004; Gruber and Müller 2002, 2005; Rugg et al. 1995), could be investigated in future studies.

These findings also match those of other studies linking the amplitude of the P2 to lexical retrieval (Fargier and Laganaro 2017, 2020; Indefrey 2011) and the ease thereof (Rabovsky et al. 2021; Strijkers et al. 2010). From the current and previous studies on contextually guided naming (Klaus et al. 2020; Piai et al. 2014; Piai, Roelofs, Rommers, & MariPiai et al. 2015a, b, 2017, 2018, 2020; Roos et al. 2023; Roos and Piai 2020), we know that participants initiate word retrieval processes before the picture appears. This is also reflected in the picture naming times of the present study across conditions, showing that pictures following constrained sentences were named significantly faster than scrambled pictures as a low-level visual control condition. If participants first had to recognize the depicted object and retrieve the respective word for it, they would need more time to name a meaningful picture than a scrambled picture. This should lead to an easily accessible target word at picture onset (as the concept has already been activated previously), and thus to a lower P2 amplitude. Unlike the P2 results for scrambled pictures discussed above, those for constrained pictures are in line with research linking the P2 to lexical selection and its effort.

#### Bare and unconstrained pictures (visually guided naming)

The VEPs of bare and unconstrained picture naming conditions did not significantly differ. This is in line with their respective naming times, which did not reveal any differences between these two conditions either. Both conditions show pictures of normal objects and neither their VEP nor naming times seem to be affected by whether the lead-in sentence is reversed or meaningful. This suggests that visually guided picture naming is not easily influenced by potentially distracting or non-constraining lead-in sentences, and instead remains primarily *visually guided*.

In fact, the same was observed in a previous case study of a 23-year old person with aphasia due to extensive left hemisphere damage (Chupina et al. 2022). Falling exactly into the age range of the participants of the present study, the naming results of this participant showed no difference between unconstrained and bare picture naming (without preceding reversed speech). This reinforces the idea that picture naming in unconstrained sentences is highly comparable to bare picture naming following reversed sentences, strongly suggesting that unconstrained naming is a well controlled and valid baseline contrast for constrained naming. Given the same behavioral as well as ERP outcomes for these two conditions, we discuss the potential omission of bare picture naming in future versions of the CLaP in the next section.

### Adaptations of the CLaP

The CLaP yields a lot of flexibility and can be modified to adapt to one’s preference or provide different contrasts of interest while the overall framework of conditions and trials stays the same. In the introduction we already mentioned studies arguing that time-reversed speech provides a less-optimal baseline for speech processing (Narain et al. 2003; Stoppelman et al. 2013). To counteract this issue, one could readily substitute the time-reversed speech condition with a different baseline for speech processing, such as noise vocoded speech, for example.

Another adaptation could be replacing the auditory comprehension part with reading comprehension. For this, one could use sentences presented word-by-word, as we have previously done (e.g., Roos et al. 2023; Roos and Piai 2020). Reversed speech sentences in this case could be replaced by scrambled word sentences. This version of the CLaP would then yield measures of word reading contrasted with non-word reading.

The similar outcomes of the bare and unconstrained picture naming conditions in behavioral as well as ERP results allow the possibility of omitting bare picture naming from the paradigm. In this case, however, one might lose the real effect of scrambled picture naming, as reversed speech sentences would then always be followed by scrambled pictures. Thus, participants would not have to wait for the picture after reversed sentences to appear and recognize whether it is a real or a scrambled image. Instead, they could already prepare for a scrambled picture to appear and retrieve the word necessary to name these as soon as they hear that the sentence is reversed.

At the same time, if participants are explicitly instructed that reversed sentences are *always* followed by a scrambled picture, this could lead to an expectation effect. Similarly as constrained sentences pre-activate the concept completing the sentence, reversed sentences could induce the early preparation of the stereotypical utterance for *nothing* (or the word they are instructed to use for scrambled pictures). Thus, this omission could yield a potentially interesting control contrast to constrained picture naming (see also Piai, Roelofs, Rommers, Dahlslätt, et al., 2015).

The CLaP could also readily be used with different imaging modalities than EEG. We have previously done so using a contextually driven naming task in combination with magnetoencephalography (e.g., Roos and Piai 2020) and fMRI (Roos et al. 2023). Regarding a combination of the CLaP with hemodynamic imaging methods, one might have to extend and jitter the duration of the pre-picture interval (see Roos et al. 2023). For the comprehension aspect, one could integrate over a larger time window for auditory or written sentence comprehension, instead of looking at millisecond time resolution during the sentence.

### Strengths and limitations

The CLaP presents a well-controlled framework to investigate processes of language comprehension as well as production. As all trials follow the same task instructions with a simple trial structure across conditions, we minimize the risk to capture task- or condition-specific confounds between trials. Our paradigm also includes a more naturalistic setting of language in terms of context-driven picture naming. However, being well-controlled inevitably makes it less naturalistic in terms of language use in real life. We would therefore like to stress the fact that, after all, the CLaP is merely an experimental paradigm and does not provide a complete picture of language as in conversation or other real life settings. Instead, the CLaP, in any version thereof, only allows to investigate those specific mechanisms of comprehension and production that one decides to include in the paradigm.

Another important limitation regards the topographies that we report here for the ERP and TFR results. We would like to emphasize that the spread of the EEG signal over the scalp as shown in our topographic plots does not directly reflect *where* in the brain the signal differences derive from. Instead, these plots primarily serve as a comparison of topographies between conditions, assuming that the signal spreads in the same way, independent of condition. Thus, the topographies we present here should be interpreted with caution, keeping these aspects in mind.

Moreover, as with many language studies, the group effects we present here might not be present in all tested subjects, and future studies could focus on examining prevalence measures instead (e.g., Ince et al. 2021). Also, for the purpose of the present study we exclusively tested this paradigm with a group of young healthy participants aged between 18 and 28 (as is commonly done in the literature), which is not a sample representative enough for generally benchmarking the effects of a new paradigm. This is especially relevant for the application with neurological populations, which in many cases tend to be above the age range of participants tested here.

Regarding the comprehension contrast of our paradigm, we would like to note that our analyses for this were rather simplistic, serving as a first step. In our own future studies using the CLaP, we are planning on investigating the comprehension part of the paradigm using source localization. As the present study did not include participant-specific MRI scans, we were not able to perform this type of analysis here. If one is interested in a more detailed investigation of the auditory sentence contrast, this could possibly be done with analysis approaches as used in naturalistic spoken language comprehension (Zioga et al. 2023).

## Conclusion

The present study introduces the CLaP as a new Concise Language Paradigm to investigate aspects of language comprehension and production within the same trial. We provided a first version of the CLaP for measures of comprehension and production characterizing the language system, here with a particular focus on spoken word production. We propose several optional adaptations to further improve the CLaP and potentially use it with different populations. The conciseness and richness, as well as the flexibility in adapting the paradigm to different versions yield promising outlooks to further investigate the relationship between language comprehension and production in neurotypical as well as neurological populations, using one’s own desired version of the CLaP framework.

## Data Availability

All data and code are available via the Donders Repository (10.34973/19gn-7v46*).*
